# Feasibility of the LvL UP digital lifestyle coaching intervention designed to prevent non-communicable diseases and common mental disorders

**DOI:** 10.1038/s41598-025-30960-z

**Published:** 2025-12-22

**Authors:** Jacqueline L. Mair, Ahmad I. Jabir, Alicia Salamanca-Sanabria, Oscar Castro, Shenglin Zheng, Roman Keller, Bea Franziska Frese, Chang Siang Lim, Aishah Alattas, Samarth Negi, Akshaye Shenoi, Rob M. van Dam, EShyong Tai, Elgar Fleisch, Florian von Wangenheim, Lorainne Tudor Car, Falk Müller-Riemenschneider, Tobias Kowatsch

**Affiliations:** 1 Future Health Technologies, Singapore-ETH Centre, Campus for Research Excellence and Technological Enterprise (CREATE), Singapore, Singapore; 2 Centre for Digital Health Interventions, Department of Management, Technology, and Economics, ETH Zurich, Zurich, Switzerland; 3 Yong Loo Lin School of Medicine, National University of Singapore, Singapore, Singapore; 4 Institute for Human Development and Potential (IHDP), Agency for Science, Research and Technology (ASTAR), Singapore, Singapore; 5 Saw Swee Hock School of Public Health, National University of Singapore and National University Health System, Singapore, Singapore; 6 Department of Exercise and Nutrition Sciences, Milken Institute School of Public Health, George Washington University, Washington, DC USA; 7 Centre for Digital Health Interventions, Institute of Technology Management, University of St. Gallen, St. Gallen, Switzerland; 8 School of Life Course and Population Sciences, King’s College London, London, UK; 9 Department of Primary Care and Public Health, School of Public Health, Imperial College London, London, UK; 10 Digital Health Center, Berlin Institute of Health, Charite University Medical Centre Berlin, Berlin, Germany; 11 Institute for Implementation Science in Health Care, University of Zurich, Zurich, Switzerland; 12 School of Medicine, University of St. Gallen, St. Gallen, Switzerland

**Keywords:** Digital health, Chatbot, Behaviour change, Lifestyle medicine, Conversational agent, Preventive health, Human behaviour, Lifestyle modification

## Abstract

LvL UP is a smartphone-based lifestyle coaching intervention aimed at improving health behaviours, mental well-being, and preventing noncommunicable diseases and common mental disorders. It features a ‘talk and tools’ approach, combining automated health literacy coaching via conversational agent with digital tools such as journaling, life hacks, and slow-paced breathing exercises. An ‘in-the-wild’ mixed-methods study was conducted in Singapore to evaluate LvL UP’s feasibility and acceptability to inform a future definitive trial. The app was available on iOS and Android from March to August 2023 and was promoted through online and offline strategies. Data collection included in-app surveys, usage metrics, and interviews, summarised using descriptive statistics and template analysis. The app was downloaded 307 times. Data from 99 active users were analysed. Most users were female and aged 21–35 years with mild to moderate vulnerabilities in physical activity, diet, and depressive symptoms. Engagement was highest during the first eight days, with 9% remaining engaged for up to 50 days. Users rated technology acceptance highly, finding the app enjoyable, easy to use, and informative. Suggested improvements included streamlined onboarding, fixing bugs, shortening dialogues, and adding rewards. The findings support LvL UP’s feasibility and have informed enhancements for future trials.

**Supplementary Information:**

The online version contains supplementary material available at 10.1038/s41598-025-30960-z.

## Introduction

The growing burden of non-communicable diseases (NCDs) and common mental disorders (CMDs), along with their associated risk factors, contribute significantly to escalating healthcare and socio-economic costs worldwide^1–3^. Addressing the prevention, management, and treatment of NCDs and CMDs requires a paradigm shift toward, holistic body-mind interventions that prioritise health-promoting behaviours, mental well-being, and overall wellness^4,5^. However, the initiation and long-term maintenance of health-promoting behaviour change remains challenging and is often adopted by only a fraction of those in need^4^.

Lifestyle coaching, which focuses on enhancing behaviours related to physical activity, nutrition, stress management, and sleep, has demonstrated its potential to improve health outcomes and reduce chronic disease risks^6^. Yet, traditional delivery models, typically involving in-person or group-based sessions with a single practitioner, are time- and resource-intensive, limiting accessibility and scalability^7^.

To address these challenges, innovative delivery methods are required, particularly those that can overcome barriers related to cost, accessibility, and sustained engagement. Advances in mobile health (mHealth) technologies and artificial intelligence present new opportunities for scalable and user-friendly interventions. Conversational agents (CAs), or chatbots, have emerged as promising tools for delivering tailored health behaviour support^8^. With their ability to engage in interactive, real-time dialogue, CAs can mimic human coaching interactions, delivering tailored advice, reminders, and motivation while being available 24/7. Despite their potential, the use of CAs in holistic body-mind lifestyle coaching, that is, addressing multiple interconnected domains related to physical and mental health, is still relatively underexplored^5^. Moreover, few CA interventions are culturally tailored to Asian population^8^.

In response to this need, we have undertaken a rigorous theory-informed and user-centred intervention development process to create “LvL UP”^9^, an app-based lifestyle coaching intervention designed specifically for the Singapore context. The intervention employs a ‘talk and tools’ approach^10^ to deliver health literacy coaching sessions via an automated CA focused on three key areas—physical activity, diet, and mental well-being—which are complemented by behavioural tools, including a journal, life hacks, and a slow-paced breathing training game.

The LvL UP intervention is set in Singapore, a highly urbanised and densely populated city-state in Southeast Asia, home to a culturally diverse population comprising mainly Chinese, Malay, and Indian ethnic groups. As a nation facing rising rates of NCDs^11^ and CMDs^12^, Singapore presents a compelling case for preventive health interventions, particularly among young adults. The country’s advanced digital infrastructure, high smartphone penetration, and strong government support for digital adoption make it an ideal setting for deploying mHealth interventions.

This study aimed to assess the technical feasibility, user satisfaction, and recruitment capability of the LvL UP intervention in a real-world setting, with the goal of informing future trials and implementation strategies.

## Methods

### Intervention development

**Fig. 1:**
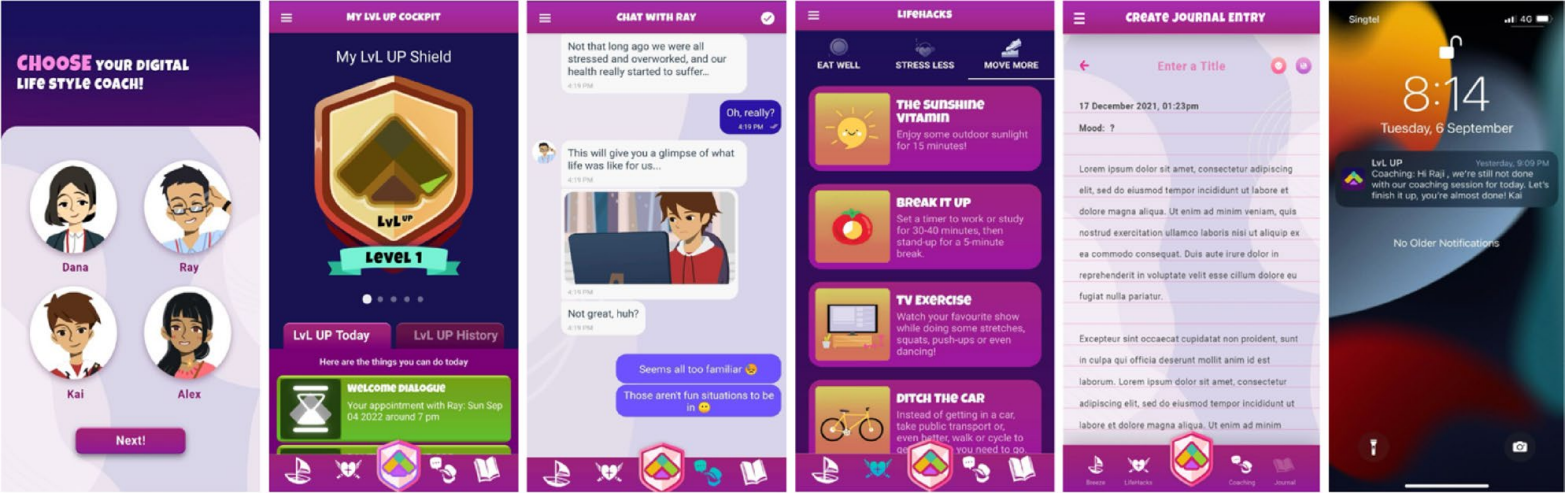
Screenshots of key LvL UP components, including conversational agent personas, home screen, chat dialogue, life hack, journal, and notifications.

The LvL UP intervention has been developed as part of a 5-year programme of research under the Future Health Technologies Programme in Singapore^13^. An iterative and user-centred development process was carried out in line with the multiphase optimization strategy^14^ and the UK Medical Research Council guidelines for developing and evaluating complex interventions^15^. Based on findings from initial market analyses^16,17^, evidence syntheses^5,18,19^ and qualitative studies with potential stakeholders^20,21^, a behavioural diagnosis was conducted using the Capability, Opportunity, Motivation, Behaviour (COM-B) model of behaviour change^22^ to develop a conceptual model underpinning the intervention^9^. We then engaged in a user-centred prototyping process to develop LvL UP version 1.0; a smartphone app comprising a motivational interviewing (MI)-inspired CA to act as a ‘digital lifestyle coach’, ‘life hacks’ to encourage healthy habit formation, and ‘tools’ supported by a transdiagnostic cognitive behavioural therapy approach including a slow-paced breathing training game and a journaling tool to promote emotional literacy. Four different CA personas were developed to represent typical Singaporean lifestyles, and users could choose one to communicate with throughout the intervention (Fig. 1). Additionally, gamification and storytelling features were built into the intervention design to encourage sustained engagement with the app. This included rewards for task completion and unlocking new features as the intervention progresses. Each of these components is described in detail elsewhere^9^.

In brief, the CA was built using an open-source platform called MobileCoach (www.mobile-coach.eu)^23^that operates rule-based with scripted sessions and pre-defined answer options developed by domain experts of the research team. The text interaction includes very minimal free-text input and the CA has no natural language processing ability which ensures the fidelity and safety of the intervention content in the health context as used in previous interventions^24–27^. Coaching sessions deliver evidence-based psychoeducational content with an MI inspired communication strategy focused on three lifestyle behaviour pillars:

- *Move more*: increasing physical activity (e.g., daily step count), exercise (home-based training), reducing sitting time/screen-based activity.
- *Eat well*: encouraging a nutrient-rich diet, reduced snacking, food purchasing and meal preparation suggestions, links between diet and well-being.
- *Stress less*: activities based on Cognitive Behavioural Therapy (CBT) evidence, including emotional regulation, behavioural activation, cognitive flexibility, and problem-solving therapy.

Life hacks are actionable health and well-being-related tips, delivered either in the morning, at lunchtime or in the evening, that can be easily implemented into the daily routine. They serve to (i) ensure a holistic approach to improving health-related behaviours regardless of the coaching pillar chosen by the participant; (ii) target improvements in self-efficacy; and (iii) facilitate routine during everyday life to encourage positive habit formation^28^. Breeze is an intervention component that uses the smartphone’s microphone to continuously detect breathing phases in semi-real time (i.e., inhale, exhale, and pause between inhale and exhale), which are then used to trigger a gamified biofeedback-guided breathing training^29,30^. Gamified biofeedback is shown as a sailing boat moving down a river which speeds up when the user performs slow-paced breathing. This targets experiential outcomes in addition to instrumental outcomes of psychological well-being and heart rate variability^31,32^. The free-text Journal tool allows users to reflect on their thoughts, feelings, behaviours, and learnings throughout the intervention and report moments of gratitude to help them reduce stress and solve problems^33^. All journal entries were stored on the device and, therefore, remained strictly confidential to the user, with only the time stamp and mood rating assigned to each journal entry being recorded on the server. Users could view and edit their journal entries at any time.

In addition to these therapeutic components, gamification, storytelling, and just-in-time intervention notifications were included as engagement strategies.

LvL UP 1.0 is intended to be used daily for 21 days, with users completing one coaching session per day, after which continued use is optional. Progress was monitored through the LvL UP Shield on the app’s home screen. Each time users completed a task in the app they received a puzzle piece to fill the LvL UP Shield. Tasks were split across four categories: Coaching Sessions, Life Hacks, Tools (Breeze or Journal), and Basics (intermediary dialogues and in-app surveys to gather evaluation data). When the shield was filled with the required puzzle pieces, users would unlock an episode from the LvL UP Storytelling approach and progress to the next level with a new shield to fill. I﻿n total, there were﻿ three levels (see Supplementary Files 1 and 2).

### Study design

Following best practice in intervention development and evaluation^14,15^, we conducted a mixed-methods, one-group feasibility study in real-world conditions to evaluate the acceptability of LvL UP 1.0 and refine components, recruitment, and retention procedures ahead of a definitive trial. The study was conducted entirely remotely in Singapore between March and August 2023. As this was a feasibility study, a formal sample size calculation was not appropriate^34^. To ensure saturation in identifying usability issues, a minimum of 20 participants is typically required based on heuristics in usability engineering^35^, but to account for the retention challenges common in remote digital health studies^36^, we aimed to recruit 200 participants.

### Ethical approval

The Institutional Review Board of the National University of Singapore (NUS-IRB-2021-623) and the Ethics Commission of ETH Zurich (EK-2021-N-109) approved the study. The study was conducted in accordance with the Declaration of Helsinki and all participants provided written informed consent. Participants who took part in interviews gave consent for excerpts from their discussions to be quoted in study publications.

### Participant recruitment

In an attempt to address diversity issues in digital health research^37,38^, the recruitment strategy aimed to reach a demographically diverse sample of adults. Both offline and online approaches were employed to maximise outreach. Offline efforts included door-to-door and on-street flyer distribution in residential areas, complemented by six student interns distributing flyers in high-footfall locations around the university campus over a 10-day period. Online recruitment leveraged various channels, including posts on Telegram groups dedicated to research participation, electronic direct mail (EDM) marketing and social media posts via the research centre’s mailing list and social platforms, and ad campaigns through a dedicated LvL UP social media page. Social media ad campaigns on Meta and Google Ads began in March 2023 for iOS and expanded to both iOS and Android from May to July 2023. Campaigns targeted English-speaking adults aged 21 and older living in Singapore. Initial ad variations were tested using Meta’s A/B testing feature, comparing a video post and a static image with positive framing (“Do you want to improve your well-being?”) against negative framing (“Are you struggling with your mental and physical well-being?”) (see Supplementary File 2). Over seven days, the positively framed ads outperformed the negatively framed ones, with a lower cost per click (S$0.34 vs. S$0.41), prompting their use for the remainder of the campaign. Additionally, a project website provided detailed information about the LvL UP app, the research program and study team, and a link to request an offline version of the LvL UP life hacks and journal template. To further extend reach, a referral link within the app enabled participants to invite friends and colleagues to download and engage with the app.

### Procedure

On opening the app, participants were prompted to register with an email address and complete an e-consent to participate in the study. They then proceeded to choose a digital coach (CA); one of four coaching personas designed to represent the target group of young and middle-aged adults (see Supplementary File 2). Participants completed a ‘Welcome Day’ dialogue which provided an overview of the goals and intended use of the intervention, a vulnerability assessment, and a shared decision-making process on which pillar (Move More, Eat Well, or Stress Less) to begin with. When the vulnerability assessment identified someone as having severe depression or anxiety (PHQ-4 score > 9) they were advised to seek mental health resources in Singapore (e.g., SOS Samaritans) and face-to-face counselling from a health professional. They were not prohibited from using the app or continuing with the study. Still, they were advised that the LvL UP intervention is designed to prevent disease and not treat a pre-existing condition. After completing the ‘Welcome Day’ dialogue, participants were free to use the app as they wished, although they were prompted to complete a coaching session and a life hack daily through the app’s home screen and in-app notifications.

### Outcome measures

Socio-demographic characteristics, including age group, gender, ethnicity, educational level, marital status, and household income were obtained via in-app questionnaires. Health status was also measured by the single-item Self-Rated Health Index^39^ and the World Health Organization-Five Well-Being Index (WHO-5)^40^. The health vulnerability assessment included the Patient Health Questionnaire-4 (PHQ-4) for symptoms of depression and anxiety^41^, the International Physical Activity Questionnaire (short form)^42^ and a dietary guideline adherence questionnaire developed for this study based on Singapore’s My Healthy Plate (see Supplementary File 2).

The efficacy of each recruitment channel was assessed using dynamic link tracking and QR codes in all promotional materials.

The feasibility of the LvL UP app was assessed by (i) user engagement in terms of app usage (i.e., response to notifications, timestamps and duration of app component use, and app usage patterns), (ii) session alliance with the CA, self-reported on a six-point Likert scale anchored from 0 (‘not at all’) to 5 (‘completely)^43^, and (iii) technology acceptance, including perceived enjoyment, ease of use, usefulness, and control, self-reported on a six-point Likert scale anchored from 0 (‘strongly disagree’) to 5 (‘strongly agree’)^44^ (see Supplementary File 2).

User satisfaction with LvL UP was assessed using a short feedback survey containing two open-response questions that asked participants what they liked about the app and what needed to be improved. Additionally, the cultural relevance of the app content was assessed across three dimensions of equivalence: functional (whether it contains familiar behaviours, relevant cultural context, and culturally tailored goals and examples), conceptual (whether the underlying ideas and expressions are meaningful to the target. group), and linguistic (whether the language, including regional variations, idioms, and slang, is appropriate)^45^. These were rated on a six-point Likert scale ranging from ‘not at all’ (0) to ‘completely’ (5). For those completing the short feedback survey, an option to participate in an interview was offered. Depending on participant preference, interviews were conducted virtually using Zoom or WhatsApp chat by two members of the research team (AIJ and BF). The topic guide for the interview focused on motivation to use the app, experience using the app’s features, and perceived impact of the app. It was piloted with members of the study team and consisted of semi-structured, open-ended questions, with more specific probes used to solicit additional information (funnelling technique).

After completing at least one coaching session, all self-report surveys were available in the app and tied to the LvL UP Shield progress to encourage completion. Participants were reimbursed with a S$5 shopping e-voucher upon completing the short feedback survey and a S$20 shopping e-voucher for interviews.

### Data analysis

We used Firebase Analytics to analyse dynamic links and summarise installs from each recruitment channel. Data from the Google and Meta Ads Manager were used to summarise ad performance including impressions, click through rate, cost per click, link clicks, and ad spend.

Vulnerability assessment cut-off scores were established as follows: PHQ-4 score = 3 (indicating symptoms of anxiety and depression); IPAQ-short form total physical activity in mins per week =  < 120 (below physical activity guidelines); dietary guideline adherence score = 18 (not consuming recommended portions of key food groups).

App usage data were analysed using R (version 4.4.2) and Jupyter Notebook. Quantitative survey data were summarised using descriptive statistics and open-ended responses were coded and analysed together with the interview data by one researcher (JLM). Data are reported as mean ± standard deviation throughout unless otherwise specified.

Interviews were automatically transcribed verbatim by the Zoom platform or downloaded directly from WhatsApp. Two members of the research team (AIJ and XL) reviewed the transcripts for errors and de-identified the files. Transcripts were then uploaded to Atlas.ti (V.9) for analysis. Template analysis, a form of thematic analysis, was used to analyse the data^46^. The initial template (codebook) was defined a priori and covered four key domains based on our research aims (i.e. to improve the LvL UP app): (i) participants’ perceptions of the app, including coaching sessions, life hacks, the journal, Breeze, storytelling (animations), gamification (Shield), in-app notifications, and overall look and feel; (ii) suggestions for improvement; (iii) barriers and facilitators to app use; and (iv) perceived impact on health and behaviour. To ensure rigor in the coding process, two researchers (AIJ and SZ) independently coded an equal subset of transcripts using a deductive approach. Each researcher maintained a reflective notebook to document emerging codes and analytical insights. Following independent coding, they reviewed each other’s transcripts, compared codes, and discussed any discrepancies or additional codes that emerged beyond the initial template. Disagreements in coding or code definitions were resolved through discussion, with a third reviewer (JLM) acting as an arbiter when consensus could not be reached. The codebook was iteratively refined until all coders reached an agreement. The template was then applied to the open-ended survey responses by JLM. The final template covered three overarching topics: (i) app components, including coaching sessions, life hacks, Breeze, and the journal; (ii) engagement strategies; and (iii) overall user experience.

## Results

### Reach

The average click through rate across all social media ad campaigns was approximately 1%, which is considered reasonable for social media news feed ads^47^. While EDM achieved the highest click through rate, friend referrals proved most effective in converting clicks into app installs (Table 1). A technical issue with link tracking on Meta prevented the accurate tracking of app installs through that platform.

**Table 1:** Recruitment channel analysis.

Channel	Type	Impressions	Clicks	Click through rate (%)	Installs	Install rate (%)	Spend (S$)	Cost per install (S$)
Google	Ad campaign	233,667	2220	0.95	219	9.87	1,131.31	5.17
Facebook and instagram	Ad campaign	107,528	1042	0.97	0	N/A	637.87	N/A
LinkedIn, twitter and facebook	Research centre’s page post	904	29	3.21	1	3.45	0.00	0.00
Telegram channel	Posts on #SGResearchLobang and #paidstudiesNTU	4600	704	15.30	44	6.25	0.00	0.00
E-Mail	Research Centre’s Mailing list	220	145	66.00	4	2.76	0.00	0.00
Flyer distribution	University campus (student interns)	2000	171	8.55	32	18.71	1,244.07	113.10
Flyer distribution	Residential Area (Little Ants)	10,100	5	0.05	0	0	1,668.00	N/A
Friend referral	Link sharing	unknown	3	N/A	2	66.67	0.00	0.00

### Recruitment

In total, 307 individuals downloaded the app, 249 opened the app and registered an email address, 215 verified their email address, 204 started the welcome dialogue, 105 completed the vulnerability assessment, and 99 (herein defined as ‘active users’) completed the ‘Welcome Day’ dialogue to unlock the coaching sessions. The recruitment cost per install was S$15.92 and per active user was approximately S$47.29. The main reasons people download the app were (1) to see what the app was about (n = 52; 41.6%), (2) to improve overall well-being (n = 36, 28.8%), (3) to lose weight (n = 13; 10.4%), (4) to learn about healthy living (n = 7; 5.6%), (5) to get more active (n = 5; 4.0%), (6) to improve diet (n = 3; 2.4%) and (7) to manage emotions (n = 3; 2,4%).

The active users were mostly female, aged 21–35 years, of Chinese ethnicity, single, educated to university degree level, and in employment (Table 2), with mild to moderate health vulnerabilities in terms of physical activity, diet, and mental well-being (Table 3). Of the 19 active users completing the health status survey, average mental well-being, (measured by the WHO-5) was rated 16.76 out of 25 (67.05%) and the majority self-rated their health as good (Table 2).

**Table 2:** Participant demographic profile.

	Total n (%)
Age (years) (N = 99) 21–35 36–50 51 + Prefer not to say	69 (69.7%) 21 (21.2%) 6 (6%) 3 (3%)
Sex (N = 99) Female Male Prefer not to say	63 (63.6%) 33 (33.3%) 3 (3%)
Ethnicity (N = 99) Chinese Indian Malay Other Prefer not to say	74 (74.7%) 6 (6.1%) 2 (2.0%) 12 (12.1%) 5 (5.1%)
Education level (N = 99) University degree Diploma A-level O-level/N-level Prefer not to say	68 (68.7%) 15 (15.1%) 6 (6.1%) 4 (4.0%) 6 (6.1%)
Monthly household income (S$) (N = 63) < 2,999 3,000–6,999 7,000–10,999 > = 11,000 Prefer not to say unknown	6 (10.0%) 17 (27.0%) 14 (23.0%) 7 (11.0%) 16 (25%) 3 (5.0%)
Employment status (N = 63) Employed Student Homemaker Unemployed Retired Prefer not to say	38 (61.0%) 12 (19.0%) 3 (5.0%) 4 (7.0%) 1 (2.0%) 3 (5.0%)
Marital status (N = 63) single Married Divorced or separated Prefer not to say	38 (60.0%) 18 (29.0%) 2 (3.0%) 5 (8.0%)
Self-rated health (N = 19) Excellent Very good Good Fair Poor	0 (0.0%) 6 (31.6%) 12 (63.2%) 1 (5.3%) 0 (0.0%)

**Table 3:** Participant vulnerability.

	Total n (%)
Mental health vulnerability score (N = 99) No risk Low risk Moderate risk High risk Very high risk	20 (20.2%) 22 (22.2%) 40 (40.4%) 9 (9.1%) 8 (8.1%)
Diet vulnerability score (N = 99) No risk Low risk Moderate risk High risk Very high risk	56 (56.4%) 43 (43.4%) 0 (0.0%) 0 (0.0%) 0 (0.0%)
Physical activity vulnerability score (N = 99) No risk Low risk Moderate risk High risk	62 (62.6%) 8 (8.1%) 5 (5.1%) 24 (24.2%)

### Technical feasibility

Technology acceptance was rated good overall, with most people agreeing the app was enjoyable, easy to use, and provided useful information (n = 31; mean ± SD; 3.73 ± 1.07). The cultural relevance of the app content was rated moderate (n = 10; functional equivalence 3.6 ± 0.97; conceptual equivalence 3.6 ± 0.97; and linguistic equivalence 3.5 ± 1.18) and users’ session alliance ratings with the CA were good (n = 19; 3.9 ± 1.22).

**Fig. 2:**
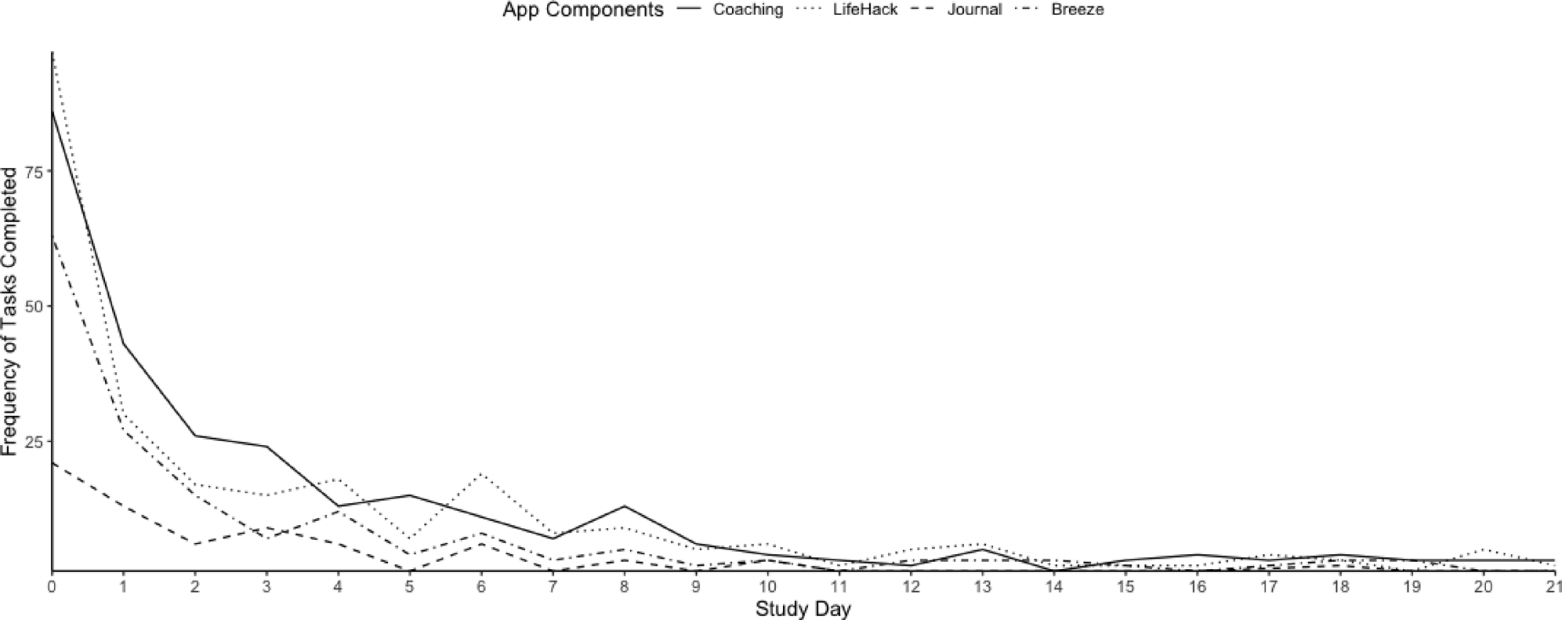
LvL UP app component usage since installation.

**Fig. 3:**
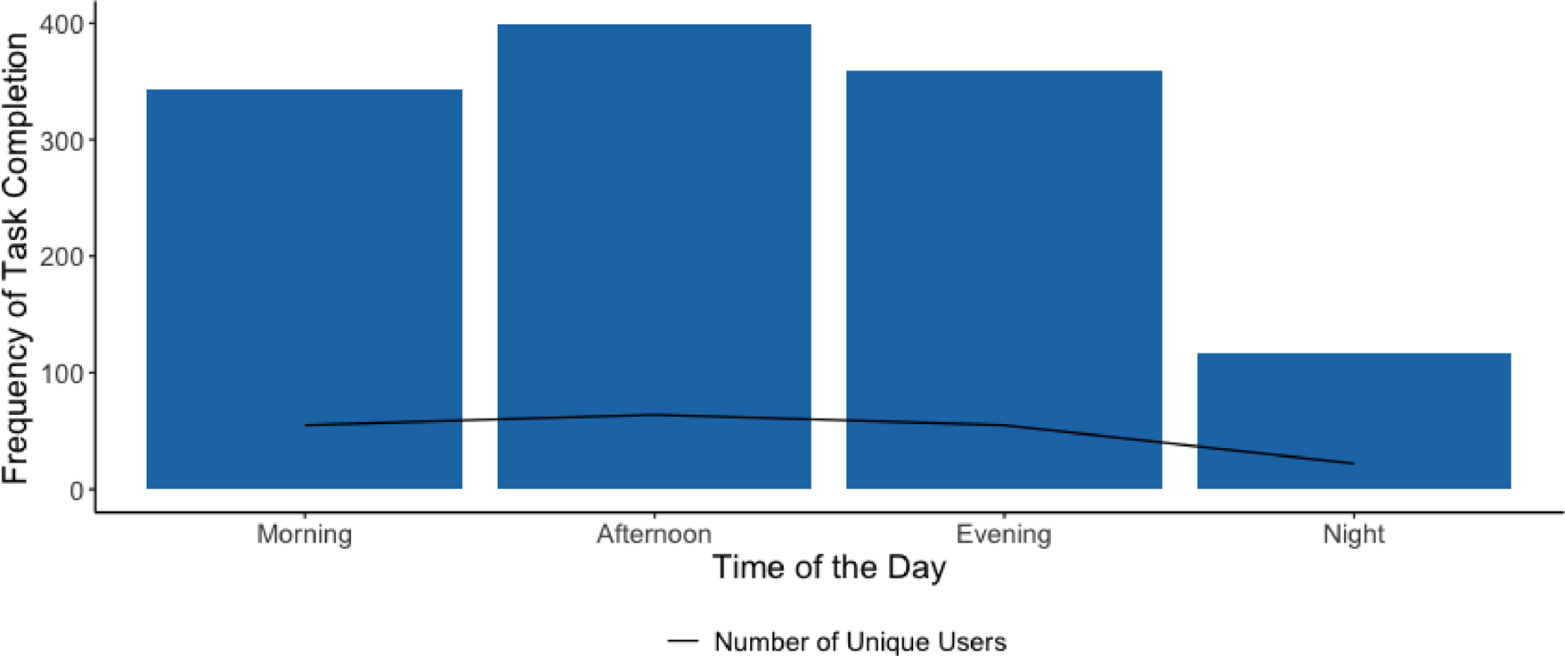
Number of tasks completed per time of day. *Note*. Morning = 6am–12 pm, Afternoon = 12–6 pm, Evening = 6 pm–12am, Night = 12–6am.

**Fig. 4:**
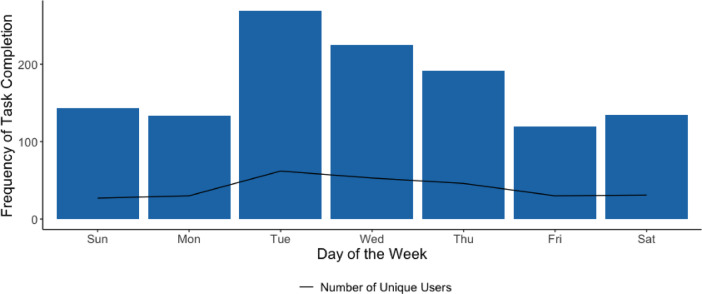
Number of tasks completed per day of the week.

Daily engagement with the LvL UP app features (coaching sessions, life hacks, journal, and Breeze) since installation is summarised in Fig. 2. Usage was highest in the first eight days*.* By week two, 77.8% (77/99) of the initial users were lost to attrition. Of those who spent more than one day on the app, they completed an app activity approximately every three days (3.21 ± 7.23 days). Users were most active in the afternoons (Fig. 3) and on Tuesdays (Fig. 4).

On average, active users completed three coaching sessions (mean ± SD; 3.35 ± 4.53), five life hacks (4.92 ± 6.52), two journal entries (2.34 ± 2.30) and four slow-paced breathing training sessions (4.29 ± 4.09). Active users spent between 1 and 28 days (4.07 ± 5.02 days) completing at least one task on the app and 35.4% (35/99) were active for less than one day. Among the active users, 9.1% (9/99) completed Level 1, 4.0% (4/99) progressed to Level 2, and 3.0% (3/99) completed the intervention. Only one user managed to complete the intervention within 21 days. Five users (5%) were considered super users, engaging in the intervention regularly for 21 days or longer.

A total of 405 coaching session notifications were successfully delivered. Of those, 77.8% (315/405) were responded to, and 28.9% (117/405) were within 10 min (1.53 ± 2.41 min). The median response time between notification delivery and observed coaching session engagement was 57 min. However, the notification response rate could be higher as not all activities on the app were tracked (i.e. the time user opened the app) due to system limitations.

### Acceptability

Fifty participants completed the short feedback survey, and 14 interviews were conducted (n = 11 on Zoom and n = 3 on WhatsApp chat). The interviews took, on average, 33 min (15.8–50.0 min). Feedback was centred around the positive and negative aspects of (i) the app components, including the coaching sessions, life hacks, Breeze, and journal, (ii) engagement strategies, and (iii) overall user experience.

#### LvL UP intervention and app components

Many participants had positive experiences with the CA and the coaching sessions, describing the content as helpful, comprehensive, and interactive. Some mentioned that the intervention provided support and served as a reminder to make healthier choices in everyday life:

*“It had a lot of tips on how to eat well, and not just like cooking wise. I was quite impressed with the social situation one, because yeah, I did really think about how the social situation would affect our eating habits a lot. And then in particular…communicating how to say no in a way that doesn’t affect the other party.” [Interviewer: like, how to reject the food?] “Yeah…that stood out for me*.” (ID09, female, 21–35 years old)

“*I think LvL UP serves as an accountability partner in terms of your well-being journey because you are required to check in every day. And if you don’t then [it will] give you a notification. So, I think there is a good reminder that you have to be mindful of these new habits to incorporate and learn new things every day.”* (ID08, female, 21–35 years old)

*“It helps that I don’t feel alone when I don’t have a friend to work out with. And I don’t get judged by others. I felt I looked forward to communicating with Kai [CA] again and he is very personal despite it being a chatbot only*. (ID12, female, 36-50 years old)

Some felt the content was personalised which made it more motivating, while others felt the content could be more relevant to their needs.

*“I like the chatbot, it makes it personalised and creates more motivation with the regular check ins and all. The exercises and concepts discussed via chatbot are comprehensive and second only to live discussions with coaches, I feel.”* (ID13, female, 21–35 years old)

On the other hand, many participants mentioned that the coaching sessions were too long and responses from the CA were too slow, and that daily coaching may be too demanding. Additionally, elements of the MI style of coaching did not seem to work well in a chat-based format, for example, asking for permission before progressing with advice.

*“….they [CA] would pose a question and then I’ll have to click ‘yes, I want to hear more’, before they put the next topic in. Well, personally, I feel that if you just put the topic itself and carry on talking, without putting in the random interjecting statements like, ‘yes, I want to hear more’, it’s a bit easier for me to process the information because it comes to me directly. I do not need to keep on clicking the button*.” (ID07, male, 21–35 years old)

*“I think it’s really cool that there’s the badges that you get to earn. The icon that I was talking about when you complete an activity. So, you get to see your progress. That is something that resonates with me because I’m somebody that really likes to see gamification integrated into whatever I’m doing, because it just makes the overall experience much more enjoyable, much more fun.” (ID02, male, 21–35 years old)*

#### Engagement strategies

There was some appreciation for the storytelling approach which participants found interesting and potentially effective in keeping people engaged, but it was also perceived as time consuming.

*“…the storyline thing…the characters that you can choose, and they have the daily check-in with you. So, it feels like a game but not really a game where you can follow the character’s development*…” (ID09, female, 21–35 years old)

*“LvL UP tries to give you a story. I mean, it’s not bad lah, the story, because maybe you identify with a certain character like the lady, Dana, who drinks bubble tea and whatever, right? But when I’m pressed for time, then it becomes very irritating.”* (ID06, female, 36–50 years old)

Many participants talked about the need for incentives and rewards, tied to the gamification features of the app, to encourage its continued use as well as motivate health behaviour change.

*“I think incentive-based health care provision is actually quite effective. Like anecdotally, I’ve seen people going to lengths walking their 10,000 steps a day to clock their Healthy365 [step counting app] steps, you know, so that they can get rewards. Yeah, so I do think that the use of incentives might motivate people to pursue a healthier lifestyle*.” (ID07, male, 21–35 years old)

*“Perhaps once you manage to accumulate all these icons, then you get some sort of monetary incentives or something like that. I think that would really incentivize people to want to do the tasks.”* (ID02, male, 21–35 years old)

*“Maybe you can connect with other partners like merchants to give discount vouchers, or like you can collaborate with I don’t know, like speaker or gym source to provide more services or something. (*ID08, female, 21–35 years old)

#### Overall user experience

The LvL UP app was generally described as easy to use although some participants mentioned that there were too many components to the app and the levelling up criteria was not always clear. There was a preference for straightforward functionality, the option to tailor settings in the app (such as timing of coaching sessions), and an app that required minimal time investment.

*“The ability to schedule our coaching sessions was a good move to help integrate it into my lifestyle.”* (ID14, male, 21-35 years old)

*“…because Lumihealth is kind of like touch and go once you see the quest, then you pretty much know what to do. As for LvL UP, for the most part I would have to stay on the app for maybe let’s say, 10 to 15 min before I get the task done.”* (ID02, male, 21–35 years old)

Regarding interactions with the CA, the predefined answer options were generally appreciated from an ease of use perspective, however participants would prefer more autonomy over their responses, through either more diverse answer options or the ability to type their own response.

*“Offer more options to disagree with the coach. I’m going through the Move More pillar, and there have been various instances where I had to select an option equivalent to ‘Yes’ of varying degrees to agree with the chatbot because there is no negative option, even though I do not agree with the statement put forth. The conversation then ends up feeling shallow, like not true to self or lying.” (WA03, female, 36*–*50 years old)*

*“But sometimes I think I am just very lazy to type, lah. That’s good to have an option. But I think it can be another option to let you type yourself”. [Interviewer: So, having a variety of options to respond to the coach is something that you would prefer. Am I right to say that?] “Yeah, correct.” (ID05, female, 21*–*35 years old)*

Feedback on the user interface was mixed; some liked the colourful look and feel while others said a cleaner interface would be preferable. The animated design of the digital coaches and the story were sometimes described as “childish”, with one participant suggesting that videos of real people would be preferred.

There was an expectation that the app would better integrate with existing apps and devices for a more personalised experience.

*“Also, consider syncing with wearable devices, allow data sharing or integrate with health-related apps to have a comprehensive experience. (ID15, female, 36-50 years old)*

Finally, suggested improvements were related to usability (improving the registration process and the speed of the app, fixing bugs and app crashes).

## Discussion

This study provides insights into the feasibility and acceptability of the LvL UP 1.0 intervention delivered to adults in Singapore. The findings align with broader trends in mHealth research, highlighting both the potential and challenges of digital behavioural interventions, particularly those delivered via CAs. LvL UP 1.0 was technically feasible; all intervention components were successfully delivered, technology acceptance and session alliance were rated positively, and notifications were delivered as planned. However, active users were not representative of the broader target population, and engagement beyond eight days was low, possibly due to shortcomings in the overall user experience.

Recruitment and engagement strategies play a crucial role in the success of remotely delivered mHealth interventions. In line with previous studies^48^, we found that positively framed health messages were more effective in promoting engagement than fear-based messaging. The overall click through rate of approximately 1% was consistent with industry standards for social media advertising^47^, but conversion from advertisement clicks to active app users remained a challenge. Friend referrals were the most effective in converting interest into active participation, supporting existing evidence that peer recommendations enhance trust and motivation in digital health adoption^38^.

The demographic profile of active users in this study suggests that LvL UP appealed primarily to young, well-educated, employed women with mild to moderate health vulnerabilities. This is consistent with broader digital health trends and underscores the ongoing challenge of reaching individuals from lower socioeconomic backgrounds or ethnic minority groups^49,50^, who may stand to benefit the most from such interventions. Expanding recruitment strategies to include community-based outreach and partnerships with trusted local organizations could enhance engagement among those at higher risk of developing NCDs or CMDs but who may not (yet) be actively seeking support^38^. Additionally, while LvL UP attracted initial interest, the attrition rate—from app downloads to active participation—suggests potential barriers during early engagement, including digital literacy, perceived relevance, and trust^38^. The application of user-centered design principles in LvL UP provides a strong foundation, and future iterations can build on this by incorporating additional accessibility and culturally adapted features to ensure inclusivity and sustained engagement.

Challenges with uptake and attrition may have been exacerbated by the study’s fully remote, ‘in-the-wild’ design, where users received no researcher-led guidance beyond the app’s welcome dialogue. While this approach enhanced external validity by simulating a real-world app launch, sustained usage was low, despite the reported positive experiences with the LvL UP content. Although the usage patterns align with trends seen in other app-based^51^ and CA interventions^52^, the drop in adherence presents challenges for evaluation the therapeutic effectiveness of remotely delivered interventions. User experience, gamification, progress tracking, and rewards are key features that can enhance user retention in mHealth interventions^18,38^. Therefore, future iterations of LvL UP could explore simplifying navigation, and offering clearer onboarding guidance, feedback on activities, and achievement-based rewards, to help improve usability and retention.

Solving the problem of attrition in mHealth is an ongoing effort. However, findings from this study point to three potential solutions: (i) access to human support for those who need it, (ii) more personalised support to improve therapeutic alliance, and (iii) delivering relevant intervention support at the most optimal moments.

Firstly, adherence to mHealth interventions improves when usage expectations are clearly communicated, and human support is available^18^. Participants in this study emphasized the importance of a straightforward user experience in creating positive first impressions and the need for clear guidance on the intended use of the intervention. Offering a human connection during onboarding and throughout the intervention may foster supportive accountability^53^ and sustained engagement. Embedding tools like LvL UP within existing healthcare or community support systems, such as primary care, mental health services, or peer-led community programmes, could strengthen this accountability while offering additional opportunities for engagement. For example, a health professional or community worker introducing the app, guiding the initial setup, and providing periodic encouragement may bridge the gap between digital and human support. This blended model could enhance retention, particularly for users with greater needs or lower digital confidence. Nevertheless, relying on human involvement at scale may be impractical, and further research into optimising when and how to provide such support within mHealth interventions is needed^54^.

Secondly, as shown elsewhere, there was a clear expectation and appreciation for personalised health behaviour support^18,20^. The CA in the LvL UP app is based on a rule-based system whereby coaching sessions are predefined based on evidence-based content, and personalization is achieved through branching. While this approach ensures fidelity of the content and offers a more interactive delivery of health literacy support, branching capability is limited and it is impossible to adapt content to an individual’s changing needs. Furthermore, delivering MI in a chat-based format with pre-defined answer options is challenging because MI relies on reflecting one’s own words. In this study, attempts to “ask, offer, ask” when providing advice or guidance were also perceived as annoying. Moving forward, strategies employing large language models to personalise the interaction further using more natural language while adhering to the spirit and principles of MI may offer a solution^55^.

Thirdly, the engagement data suggest that LvL UP was most frequently used in the afternoons and on Tuesdays, which could inform future ‘just-in-time’ strategies by tailoring intervention delivery to peak usage periods when people are most receptive^56^. Recent research has shown the potential for machine learning approaches to predict these moments based on features such as device usage, phone battery level, location, and physical activity^57^. However, more research is needed in the context of complex health behaviour interventions such as LvL UP.

### Recommendations for future work

This study represents the feasibility phase of the LvL UP intervention development and was therefore not designed or powered to assess effectiveness or cost-effectiveness. In accordance with best practice in intervention development and evaluation^14,15^, these outcomes will be evaluated in a subsequent definitive trial.

The findings from this study highlight several key considerations for future iterations of LvL UP:

1. Expand and diversify recruitment strategies by complementing social media advertising with peer referral systems, community-based outreach, and partnerships with trusted local organisations. Clearer value propositions tailored to diverse user groups may also improve conversion from interest to active use.
2. Embed LvL UP within existing care and community ecosystems to enhance supportive accountability and engagement. Future iterations could explore blended models where LvL UP is introduced and supported by healthcare providers, community health workers, or peer mentors.
3. Leverage machine learning and just-in-time adaptive interventions to deliver support during periods of peak user receptivity, informed by real-world usage data and contextual cues such as time of day, activity level, and device interaction.
4. Enhance the CA experience by improving dialogue flow and incorporating hybrid interaction models—combining structured choices with optional free-text input—to allow more natural, personalised, and engaging conversations.
5. Refine onboarding and retention strategies to create good first impressions by streamlining the initial user journey and introducing elements such as progress tracking, social or peer-based accountability, and achievement-based rewards to promote sustained engagement.

## Conclusions

While LvL UP demonstrated feasibility and acceptability, engagement challenges highlight the need for further optimization. The findings align with broader trends in mHealth research, reinforcing the importance of personalization, human coaching support and accountability, gamification, and rewards in sustaining long-term engagement. Future research should explore how to balance scalability with tailored support, leveraging emerging technologies such as large language models to enhance personalisation and engagement, and subsequently, intervention effectiveness.

## Supplementary Information

Below is the link to the electronic supplementary material.

Supplementary Material 1

Supplementary Material 2

## Data Availability

The datasets collected and analysed in the current study are available in anonymised form from the corresponding author on reasonable request.

## Code availability

Code is available on OSF: https://osf.io/895cn/files.
