# Supplementary material for: Feasibility of the LvL UP digital lifestyle coaching intervention designed to prevent non-communicable diseases and common mental disorders
Source: Sci Rep. 2025 Dec 22;16:1243. doi: 10.1038/s41598-025-30960-z (PMC12789693; doi:10.1038/s41598-025-30960-z)
Supplement: Supplementary file 1 — Supplementary Material 1 [file 41598_2025_30960_MOESM1_ESM.docx]

**Supplementary File**

1. Recruitment campaign for Meta depicting (i) positive framing and (ii) negative framing.

(i)


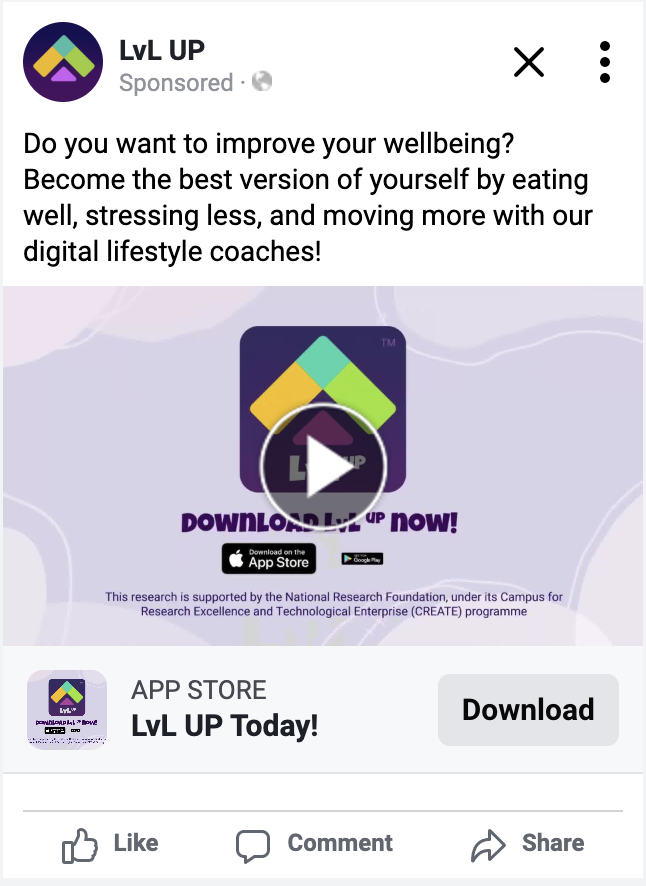

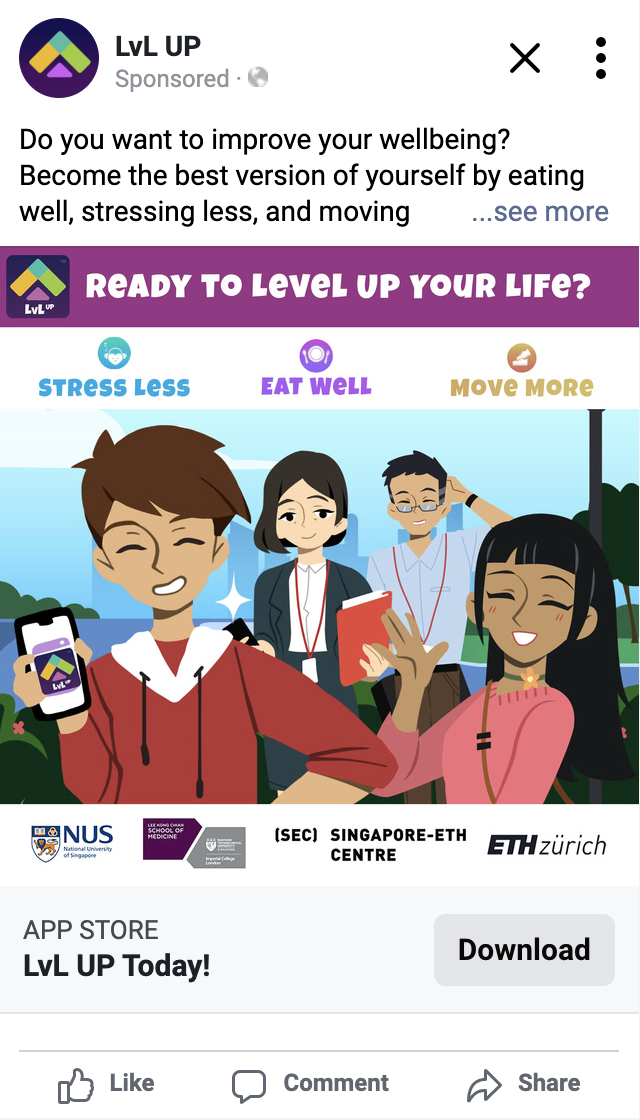


(ii)


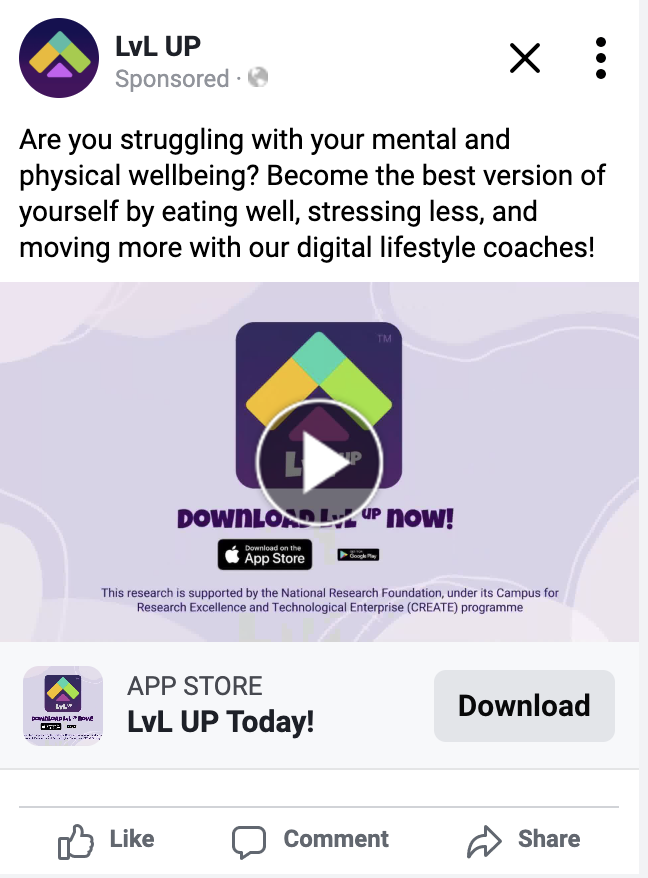

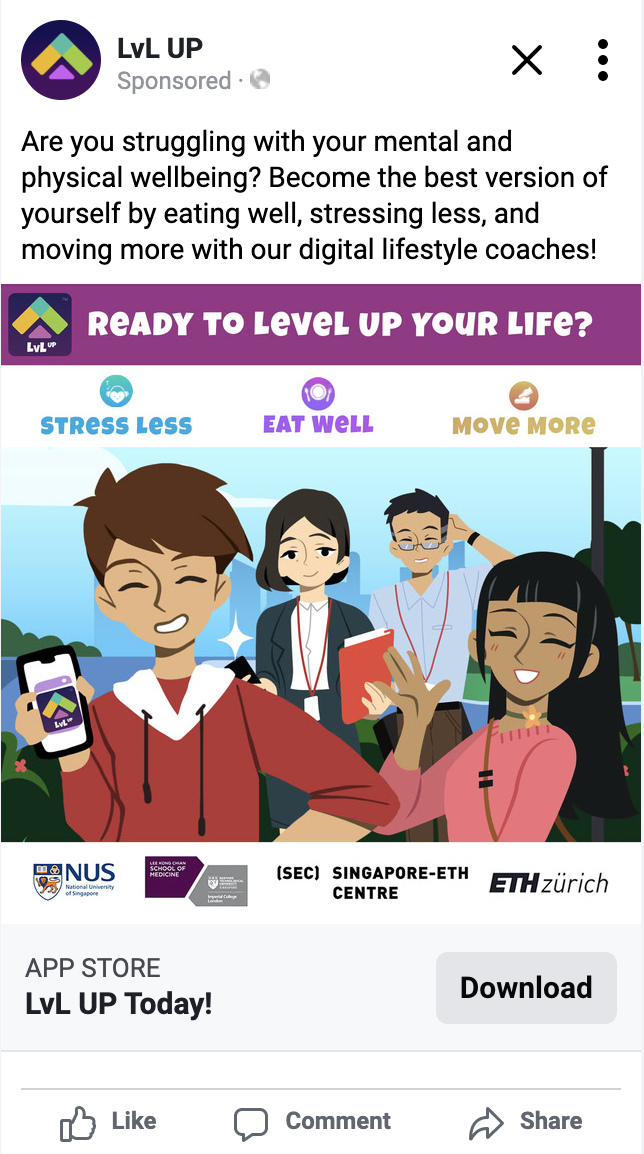


1. App onboarding screens showing the four digital coaches to choose from.


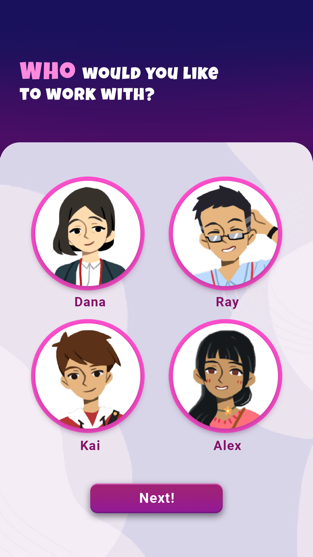

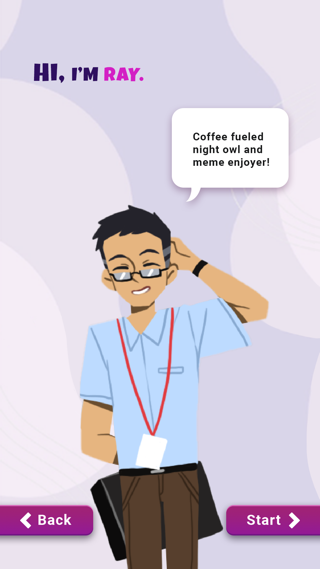

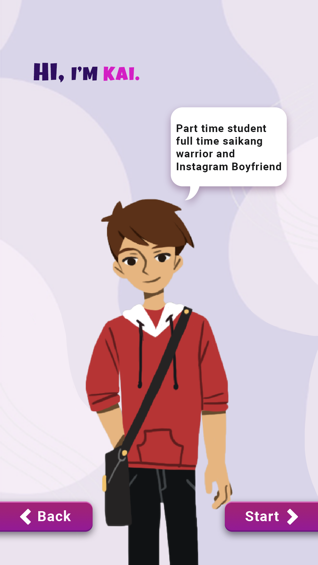

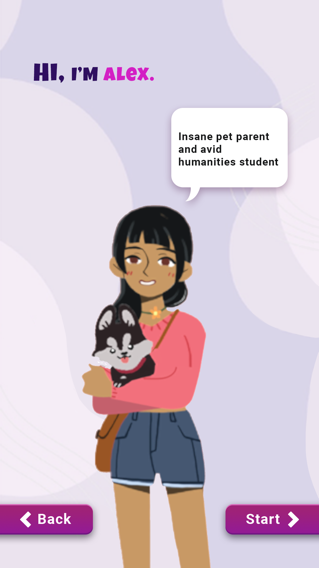

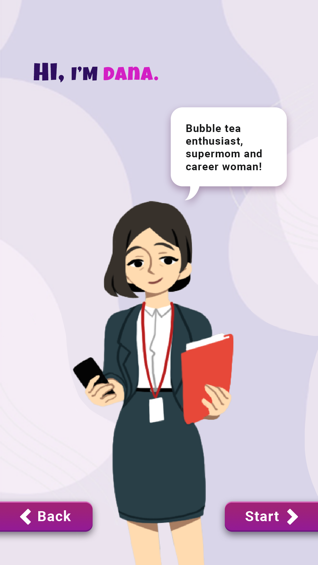


1. Leveling up criteria

| **Level** | **Task Type** | **Tasks needed for progression** | **Puzzle Piece award criteria** | **Award** | **Progress to** |
| --- | --- | --- | --- | --- | --- |
| Level 1 | LvL UP Coaching Sessions | Complete any 6 coaching sessions with Chatbot | Completion of unique pillar-specific coaching session | 1 puzzle piece/coaching session (yellow) | Level 2 upon collecting all 14 puzzle pieces (animation of new shield + LvL UP Episode 2 unlocked) |
|  | LvL UP Basics | Download LvL UP App | Download LvL UP from App Store | 1 puzzle piece (light green) |  |
|  |  | Complete “Welcome Dialogue” | Complete “Welcome Dialogue” which includes pre-test “Vulnerability Assessment” | 1 puzzle piece (light green) |  |
|  |  | Complete “About Me (Part 1)” survey via Limesurvey | Complete demographic questionnaire on Limesurvey | 1 puzzle piece (light green) |  |
|  |  | Complete “LvL UP Booklet” interest check survey | Complete “LvL UP Booklet interest check” on Limesurvey | 1 puzzle piece (light green) |  |
|  |  | Complete “Technology Acceptance” questionnaire | Complete “Technology Acceptance” questionnaire on Limesurvey (only unlocked after 1 coaching session has been completed) | 1 puzzle piece (light green) |  |
|  |  | *Complete “Check-In 1” Session | Complete “LvL 1 Review” Chatbot dialogue | 1 puzzle piece (light green) |  |
|  | LvL UP Life Hacks | Implement at least 1 life hack | Complete any one life hack activity that has not been done by user previously | 1 puzzle piece (dark green) |  |
|  | LvL UP Tools | Use Breeze or Journaling at least once | To complete 1 Breeze training session OR save 1 Journal entry | 1 puzzle piece (purple) |  |
| Level 2 | LvL UP Coaching Sessions | Complete any 6 coaching sessions with Chatbot | Completion of unique pillar-specific coaching session | 1 puzzle piece/coaching session (yellow) | Level 3 upon collecting all 14 puzzle pieces (animation of new shield + LvL UP Episode 3 unlocked) |
|  | LvL UP Basics | Complete “About Me (Part 2)” survey via Limesurvey | Complete SES questionnaire on Limesurvey | 1 puzzle piece (light green) |  |
|  |  | Complete “Working Alliance” questionnaire | Complete “Working Alliance” questionnaire on Limesurvey | 1 puzzle piece (light green) |  |
|  |  | Complete “Willingness to Pay” questionnaire | Complete “Willingness to Pay” questionnaire on Limesurvey | 1 puzzle piece (light green) |  |
|  |  | *Complete “Check-In 2” Session | Complete “LvL 2 Review” Chatbot dialogue | 1 puzzle piece (light green) |  |
|  | LvL UP Life Hacks | Implement a life hack | Complete any one life hack activity that has not been done by user previously | 1 puzzle piece (dark green) |  |
|  |  | Implement a life hack | Complete any one life hack activity that has not been done by user previously | 1 puzzle piece (dark green) |  |
|  | LvL UP Tools | Use Breeze or Journaling | Complete 1 Breeze training session OR save 1 journal entry | 1 puzzle piece (purple) |  |
|  |  | Use Breeze or Journaling | Complete 1 Breeze training session OR save 1 journal entry | 1 puzzle piece (purple) |  |
| Level 3 | LvL UP Coaching Sessions | Complete any 6 coaching sessions with Chatbot | Completion of unique pillar-specific coaching session | 1 puzzle piece/coaching session (yellow) | Final level completed upon collecting all 14 puzzle pieces (final celebratory animation + LvL UP Episode 4 unlocked) |
|  | LvL UP Basics | Complete “Cultural Adaptation” questionnaire | Complete “Cultural Adaptation” questionnaire via Limesurvey | 1 puzzle piece (light green) |  |
|  |  | *Complete “Thank You Dialogue” | Complete “Thank You Dialogue” which includes post-test “Vulnerability Assessment” | 1 puzzle piece (light green) |  |
|  | LvL UP Life Hacks | Implement a life hack | Complete any one life hack activity that has not been done by user previously | 1 puzzle piece (dark green) |  |
|  |  | Implement a life hack | Complete any one life hack activity that has not been done by user previously | 1 puzzle piece (dark green) |  |
|  |  | Implement a life hack | Complete any one life hack activity that has not been done by user previously | 1 puzzle piece (dark green) |  |
|  | ^LvL UP^ Tools | Use Breeze or Journaling | Complete 1 Breeze training session or save 1 journal entry | 1 puzzle piece (dark green) |  |
|  |  | Use Breeze or Journaling | Complete 1 Breeze training session OR save 1 journal entry | 1 puzzle piece (dark green) |  |
|  |  | Use Breeze or Journaling | Complete 1 Breeze training session OR save 1 journal entry | 1 puzzle piece (dark green) |  |

1. Vulnerability outcome measures

| ***Pillar*** | ***Variable name*** | ***Question*** | ***Answer options*** | ***Measurement Tool*** |
| --- | --- | --- | --- | --- |
| Stress Less | $PHQ1  Depression | During the last two weeks:  I have felt little interest or pleasure in doing things | Not at all:0  Several days:1  More than half the days:2 Nearly every day:3 | Patient Health Questionnaire-4 |
|  | $PHQ2  Depression | During the last two weeks:  I have been feeling down, depressed, or hopeless | Not at all:0  Several days:1  More than half the days:2 Nearly every day:3 |  |
|  | $PHQ3  Anxiety | During the last two weeks:  I have been feeling nervous, anxious or on edge | Not at all:0  Several days:1  More than half the days:2 Nearly every day:3 |  |
|  | $PHQ4  Anxiety | During the last two weeks:  I have not been able to stop or control worrying | Not at all:0  Several days:1  More than half the days:2 Nearly every day:3 |  |
| Move More | $PAIPAQVig | During the last 7 days, on how many days did you do vigorous physical  activities like heavy lifting, digging, aerobics, or fast bicycling?  ---  Enter your days below as a number:  ---  How much time did you usually spend doing vigorous physical activities on one of those days? | ___ days per week      ----  ___ hours per day  ___ minutes per day | International Physical Activity Questionnaire – Short Form (IPAQ-SF) |
|  | $PAIPAQMod | During the last 7 days, on how many days did you do moderate physical  activities like carrying light loads, bicycling at a regular pace, or doubles tennis?  Do not include walking.  ---  How much time did you usually spend doing moderate physical activities on one  of those days? | ___ days per week      ----  ___ hours per day  ___ minutes per day |  |
|  | $PAIPAQWalk | During the last 7 days, on how many days did you walk for at least 10 minutes  at a time?  ---  How much time did you usually spend walking on one of those days? | ___ days per week      ----  ___ hours per day  ___ minutes per day  Don’t know |  |
|  | $PAIPAQSB | This question is about the time spent sitting on a typical weekday in the last week. Sitting may include time spent at a desk working, chilling on the sofa, reading in a chair or lying around.  Thinking back to the last 7 days, roughly how many hours were you sitting on a typical day? | ___ hours per day  ___ minutes per day |  |
| Eat Well | $DFFFruit | How many servings of fruit do you usually eat each day? (A 'serving' is 1 medium piece or 2 small pieces of fruit or a cup of diced pieces.) This includes all fresh, dried, frozen and tinned fruit, and 100% fruit juice. | I don't eat fruit:0  1 servings or less:1  2 servings:2  3 servings:3  4 servings:4  5 servings or more:5 | Devised based on My Healthy Plate Singapore |
|  | $DFFVeg | How many servings of vegetables do you usually eat each day? (A 'serving' is half a cup of cooked vegetables or 1 cup of salad vegetables.) This includes all fresh, dried, frozen and tinned vegetables. | I don't eat vegetables:0  1 servings or less:1  2 servings:2  3 servings:3  4 servings:4  5 servings or more:5 |  |
|  | $DFFWholegrains | How many servings of wholegrains do you usually eat each day? (A 'serving' is ½ bowl of brown rice, or 2 wholemeal chapatis, or 2/3 bowl of wholegrain noodles). | I don't eat wholegrains:0  1 servings or less:1  2 servings:2  3 servings:3  4 servings:4  5 servings or more:5 |  |
|  | $DFFProtein | How many servings of protein do you usually eat each day? (A 'serving' is a palm sized piece of meat, fish or poultry, or 3 eggs, or 2 small blocks of soft beancurd). This includes poultry, red meat, fish, seafood, eggs, milk, cheese, tofu, tempeh, nuts, beans, legumes. | I don't eat wholegrains:0  1 servings or less:1  2 servings:2  3 servings:3  4 servings:4  5 servings or more:5 |  |
|  | $DFFSnack | How often do you eat potato based snacks such as potato wedges, French fries, potato chips/crisps, or other similar snacks? | Never or rarely:0  Less than once a week:1 About 1 to 2 times a week:2  About 3 to 4 times a week:3  About 5 to 6 times a week:4  About once a day:5  2 or more times a day:6 |  |
|  | $DFFFastFood | How often do you have meals or snacks, such as burgers, pizza, fried chicken, or french fries from places like McDonalds, Burger King, Pizza Hut, KFC, or other take-away food places? | Never or rarely:0  Less than once a week:1 About 1 to 2 times a week:2  About 3 to 4 times a week:3  About 5 to 6 times a week:4  About once a day:5  2 or more times a day:6 |  |
|  | $DFFSSBeverages | How often do you drink sugar sweetened beverages like Cola, chocolate drinks, Teh, iced tea, or energy drinks? | Never or rarely:0  Less than once a week:1 About 1 to 2 times a week:2  About 3 to 4 times a week:3  About 5 to 6 times a week:4  About once a day:5  2 or more times a day:6 |  |
| Wellbeing | $Wellbeing_1 | During the last two weeks:  I have felt cheerful in good spirits. | All of the time: 5  Most of the time: 4  More than half the time: 3  Less than half the time: 2  Some of the time:1  At no time:0 | WHO-5 Well-being Index |
|  | $Wellbeing_2 | During the last two weeks:  I have felt calm and relaxed. | All of the time: 5  Most of the time: 4  More than half the time: 3  Less than half the time: 2  Some of the time:1  At no time:0 |  |
|  | $Wellbeing_3 | During the last two weeks:  I have felt active and vigorous. | All of the time: 5  Most of the time: 4  More than half the time: 3  Less than half the time: 2  Some of the time:1  At no time:0 |  |
|  | $Wellbeing_4 | During the last two weeks:  I woke up feeling fresh and rested | All of the time: 5  Most of the time: 4  More than half the time: 3  Less than half the time: 2  Some of the time:1  At no time:0 |  |
|  | $Wellbeing_5 | During the last two weeks:  3 | All of the time: 5  Most of the time: 4  More than half the time: 3  Less than half the time: 2  Some of the time:1  At no time:0 |  |

1. In-app survey outcome measures

| ***Outcome*** | ***Variable*** | ***Question/Statement*** | ***Answer Options*** | ***Measurement tool*** |
| --- | --- | --- | --- | --- |
| Cultural Relevance | $CRQ1  (Functional and conceptual equivalence) | My people and cultural context are reflected in LvL UP | Not at all:0  :1  :2  :3  :4  Completely:5  Please explain your choice (Qualitative) [Optional] | Adapted Cultural Relevance Questionnaire (CRQ) |
|  | $CRQ2  (Functional and conceptual equivalence) | The goals, examples and stories presented in LvL UP are relevant to my culture | Not at all:0  :1  :2  :3  :4  Completely:5  Please explain your choice (Qualitative) [Optional] |  |
|  | $CRQ3  (Linguistic equivalence) | The LvL UP chatbot communicates using dialects and phrases (e.g., slang) that are relevant to my culture | Not at all:0  :1  :2  :3  :4  Completely:5  Please explain your choice (Qualitative) -[Optional] |  |
| Working alliance | $SAI1 | $coachName and I respect each other | Not at all:0  :1  :2  :3  :4  Completely:5 | Session Alliance Inventory (6-item WAI) |
|  | $SAI2 | I feel that $coachName appreciates me. | Not at all:0  :1  :2  :3  :4  Completely:5 |  |
|  | $SAI3 | I feel that $coachName cares about me even when I do things that they do not approve of. | Not at all:0  :1  :2  :3  :4  Completely:5 |  |
|  | $SAI4 | $coachName and I agree on what is important for me to work on. | Not at all:0  :1  :2  :3  :4  Completely:5 |  |
|  | $SAI5 | I believe the way $coachName and I are working with my problem is correct. | Not at all:0  :1  :2  :3  :4  Completely:5 |  |
|  | $SAI6 | As a result of this session with $coachName, I am clearer as to how I can change my behaviors. | Not at all:0  :1  :2  :3  :4  Completely:5 |  |
| Technology acceptance | $TAEnjoyment | I enjoy using this app | Strongly Disagree:0  :1  :2  :3  :4  Strongly Agree:5 |  |
|  | $TAEaseofuse | The app is easy to use | Strongly Disagree:0  :1  :2  :3  :4  Strongly Agree:5 |  |
|  | $TAUsefulness | The app provides useful information | Strongly Disagree:0  :1  :2  :3  :4  Strongly Agree:5 |  |
|  | $TAControl | I was able to make a lot of decisions myself in the app | Strongly Disagree:0  :1  :2  :3  :4  Strongly Agree:5 |  |

1. Feasibility Study Follow-up Interview Topic Guide
2. **What motivated you to download LvL UP initially?**

Prompts

- Was it to solve a particular problem?
- Did the app help you solve a problem that were not able to before? How so?

1. **Please describe in your own words what the LvL UP intervention was.**
2. **Describe what impact LvL UP had on your behaviours and your health**

**Prompts**

- How (or in what way) did LvL UP help you?
- Did you experience negative effects from using LvL UP?

1. **How was your overall experience using LvL UP? (e.g. coaching sessions, life hacks, breeze, journaling)**

Prompts:

- What would you change about the intervention? Why?
- How would the use of incentives (e.g. vouchers) affect your use of LvL UP?

1. **How did you find the interaction with the digital coach?**

Prompts:

- What have you learnt from the sessions with the digital coach?
- How did you find the length of the coaching sessions?
- How did you find the frequency of the coaching sessions? (making an appointment everyday)

1. **What prompted you to open up the LvL UP app when you did use it?**

Prompts:

- Where were you when you opened up the app?
- What were you doing when you opened up the app?

1. **In what ways was LvL UP relevant to your culture?**
2. **Is there anything else you’d like to share about your experience with LvL UP that we haven’t covered?**
