# Supplementary material for: Feasibility of the LvL UP digital lifestyle coaching intervention designed to prevent non-communicable diseases and common mental disorders
Source: Sci Rep. 2025 Dec 22;16:1243. doi: 10.1038/s41598-025-30960-z (PMC12789693; doi:10.1038/s41598-025-30960-z)
Supplement: Supplementary file 2 — Supplementary Material 2 [file 41598_2025_30960_MOESM2_ESM.docx]

**
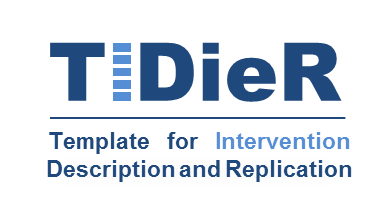
The TIDieR (Template for Intervention Description and Replication) Checklist*:**

Information to include when describing an intervention and the location of the information

| **Item number** | **Item** | **Where located** | |
| --- | --- | --- | --- |
|  |  | Page or  Appendix  number | Other (details) |
|  | **BRIEF NAME** |  |  |
| **1.** | Provide the name or a phrase that describes the intervention. | 1 |  |
|  | **WHY** |  |  |
| **2.** | Describe any rationale, theory, or goal of the elements essential to the intervention. | 2-3 |  |
|  | **WHAT** |  |  |
| **3.** | Materials: Describe any physical or informational materials used in the intervention, including those provided to participants or used in intervention delivery or in training of intervention providers. Provide information on where the materials can be accessed (e.g. online appendix, URL). | 3-5 | Additional information as online supplementary materials |
| **4.** | Procedures: Describe each of the procedures, activities, and/or processes used in the intervention, including any enabling or support activities. | 3-6 | Additional information as online supplementary materials |
|  | **WHO PROVIDED** |  |  |
| **5.** | For each category of intervention provider (e.g. psychologist, nursing assistant), describe their expertise, background and any specific training given. | n/a | The first version of LvL UP is conceptualised as a self-guided intervention and thus there is no (human) intervention provider. LvL UP has been developed by a multidisciplinary team with varied expertise, including digital health, behavioural science, mental health, cultural adaptation of digital health interventions, computer science, technology assessment, and marketing. |
|  | **HOW** |  |  |
| **6.** | Describe the modes of delivery (e.g. face-to-face or by some other mechanism, such as internet or telephone) of the intervention and whether it was provided individually or in a group. | 3-5 | The mode of delivery is primarily digital, via a smartphone app. |
|  | **WHERE** | 3-5 |  |
| **7.** | Describe the type(s) of location(s) where the intervention occurred, including any necessary infrastructure or relevant features. |  | The intervention was deployed in Singapore and was intended to be used during everyday life. |
|  | **WHEN and HOW MUCH** |  |  |
| **8.** | Describe the number of times the intervention was delivered and over what period of time including the number of sessions, their schedule, and their duration, intensity or dose. | 5 | The LvL UP intervention timeframe is intended to last approximately 3 weeks if users complete all intervention content in the suggested manner. However, users are ultimately given the autonomy to make their decision of how they would like to move through the intervention (e.g., scheduling the coaching sessions every 2 days instead of daily). |
|  | **TAILORING** |  |  |
| **9.** | If the intervention was planned to be personalised, titrated or adapted, then describe what, why, when, and how. | 3-5 |  |
|  | **MODIFICATIONS** |  |  |
| **10.** | If the intervention was modified during the course of the study, describe the changes (what, why, when, and how). | n/a |  |
|  | **HOW WELL** |  |  |
| **11.** | If intervention adherence or fidelity was assessed, describe how and by whom, and if any strategies were used to maintain or improve fidelity, describe them. | 7-8 |  |

* Hoffmann T C, Glasziou P P, Boutron I, Milne R, Perera R, Moher D et al. Better reporting of interventions: template for intervention description and replication (TIDieR) checklist and guide *BMJ*2014; 348 :g1687 doi:10.1136/bmj.g1687
